# Examining the Influence of Gender, Age, and Dominance on the Caliber of Normal Coronary Arteries in the South Indian Population

**DOI:** 10.7759/cureus.51146

**Published:** 2023-12-27

**Authors:** Anu Mohan, Asha Gopalakrishnan, Rajiv Chandran, Susan Joseph, Asha Joselet Mathew, Anjaly S Nair, Rathi Sudhakaran

**Affiliations:** 1 Anatomy, Government Medical College, Thrissur, IND; 2 Anatomy, Amrita School of Medicine, Amrita Institute of Medical Sciences and Research Centre, Amrita Vishwa Vidyapeetham, Kochi, IND; 3 Cardiology, Aster Medcity, Kochi, IND; 4 Biostatistics, Amrita School of Medicine, Amrita Institute of Medical Sciences and Research Centre, Amrita Vishwa Vidyapeetham, Kochi, IND

**Keywords:** coronary angiogram, luminal diameter, left main coronary artery, normal coronary artery, cardiac dominance

## Abstract

Background

The diameter of coronary arteries serves as a potential predictor of coronary artery diseases (CADs) that can lead to sudden death. Factors such as gender, age, and coronary artery dominance play a role in influencing the size of normal coronary arteries. The outcome of coronary interventions, to a certain extent, depends on luminal size. Given the considerable variability in luminal size within the normal population, establishing the baseline size of normal coronary arteries in a specific population can aid in estimating the severity of coronary disease and predicting the outcome of interventional procedures. The current study focuses on estimating the luminal diameter of normal coronary arteries within the context of age, gender, and cardiac dominance in the South Indian population.

Methods

A retrospective study was conducted utilizing coronary angiograms with normal findings from 453 patients, comprising 257 males and 196 females, with a mean age of 54.66±10.66 years. These patients attended the outpatient service of the Cardiology Department at Amrita Institute of Medical Sciences, Kochi, a quaternary care center, between 2015 and 2017. The luminal diameter of coronary arteries is represented as mean±SD in millimeters.

Results

In the present study, we noted that the largest coronary artery was the left main coronary artery (LMCA, 3.59±0.58 mm), followed by the left anterior descending artery (LAD, 3.50±0.52 mm), the left circumflex artery (LCX, 3.31±0.57 mm), and the right coronary artery (RCA, 3.18±0.57 mm). We further broke down the statistics to evolve a gender pattern. In the raw comparison of data, the luminal size of coronary arteries in males was greater than in females, and statistical significance was noted in all except LAD. In males, the largest coronary artery was LMCA (3.70±0.60 mm), followed by LAD (3.54±0.48 mm), LCX (3.36±0.58 mm), and RCA (3.25±0.62 mm). In females, no significant size difference was observed between LMCA (3.45±0.53 mm) and LAD (3.46±0.55 mm). Females exhibited an increase in the size of LMCA with advancing age. Regardless of right or left cardiac dominance, LMCA was consistently larger than RCA in both genders. However, in cases of co-dominance, only males demonstrated significantly larger LMCA.

Conclusion

Precise knowledge of the size of normal coronary arteries and their influence by gender, age, and dominance can be crucial for the comprehensive evaluation of CADs and the success of interventional procedures.

## Introduction

Cardiovascular mortality rates are highest in the Asian population. Statistically, compared to the Caucasian population, Indo-Asians have a 40% higher mortality rate from coronary artery disease (CAD) [1]. Statistics from India show an annual incremental increase in the prevalence of CAD [2]. The "Global Burden of Disease Study" concluded that morbidity and mortality from CAD have more than doubled in the last 30 years [3]. Industrialization, urbanization, and its ensuing lifestyle changes may be the contributing causes [4]. Despite modern techniques used for effective diagnosis and treatment of CAD, it continues to be the leading cause of morbidity and mortality in developed and developing countries [5].

Knowledge of the approximate lumen size of normal coronary arteries in males and females may be of help in the early detection of patients at risk. It may also help to choose the appropriate size of balloon or stent in interventional procedures. Estimation of the luminal diameter of normal coronary arteries has always been a topic of interest for researchers. Dhawan and Bray (1995) observed in their study that, compared to Asians, Caucasians have significantly larger vessel diameters [6]. The small size of the vessels in the Asian population can be correlated to their smaller body surface area, which in turn may lead to endothelial dysfunction and a reduction in coronary blood supply [7]. Several researchers have noted that females tend to have smaller coronary artery sizes compared to males, a factor associated with an elevated risk of mortality and morbidity following coronary artery bypass grafts (CABG) and percutaneous coronary interventions (PCI) [7]. Some researchers have noted that the postoperative mortality rate is high in patients with smaller coronary artery sizes, irrespective of gender [8].

The diameter of the coronary arteries is a potential predictor of CAD, and to an extent, the outcome of coronary intervention depends on the luminal size. Knowledge of the approximate diameter of normal coronary arteries in males and females may even help evolve gender-specific protocols for CABG and PCI, thereby improving the outcome [9]. The present study focuses on estimating the luminal diameter of normal coronary arteries and exploring the impact of gender, age, and cardiac dominance on this parameter in the South Indian population.

## Materials and methods

After obtaining clearance from the Institutional Ethical Committee of Amrita Institute of Medical Sciences and Research Centre (approval number: dissertation review/MD/MS/2015, approval date: 12/11/2015), we collected arteriographically normal consecutive coronary angiograms of 453 patients who attended the Cardiology Department of Amrita Institute of Medical Sciences, Kochi, India, between January 2015 and June 2017. The gender distribution in this sample group consisted of 257 males and 196 females, with ages ranging from 20 to 80 years. These patients underwent an evaluation for symptoms of CAD but were confirmed to be free of disease upon angiographic examination.

Coronary angiograms were performed by femoral route with Judkin or Amplatz right and left catheters. Standard projections were taken for visualization of the main epicardial coronary arteries on the Siemens Artis Zeeflat panel and Siemens Axiom Sensis XP hemodynamics imaging systems. Patients with conclusive ischemic or valvular heart diseases and tortuous vessels were excluded. The coronary angiograms selected for the study were evaluated under the guidance of a senior cardiologist. The angiographic luminal diameters of the right coronary artery (RCA) just proximal to the origin of the acute marginal artery, the left main coronary artery (LMCA) immediately proximal to its bifurcation or trifurcation, the left anterior descending (LAD) prior to its first septal branch, and the left circumflex artery (LCX) prior to the origin of its obtuse marginal branch were measured. Cardiac dominance was noted.

The male and female participants were categorized into three age groups: Group 1 (20-45 years), Group 2 (46-70 years), and Group 3 (above 70 years), with the objective of examining the impact of increasing age on coronary artery luminal diameter.

Statistical analysis

Statistical analysis was performed using SPSS Statistics version 20.0 (IBM Corp. Released 2011. IBM SPSS Statistics for Windows, Version 20.0. Armonk, NY: IBM Corp.). Categorical variables were expressed using frequency and percentage. Numerical variables were presented using the mean and standard deviation. An independent sample t-test was applied to determine the statistical significance of the diameters for gender and dominance. A paired sample t-test was used to study the statistical significance of the comparison of RCA and LMCA for dominance. An ANOVA test was used to study the statistical significance of the comparison of RCA, LMCA, LAD, and LCX diameters across age groups for males and females separately. The Bonferroni test was used to identify the significant pair. The level of significance was set at 5%.

## Results

**Figure 1:**
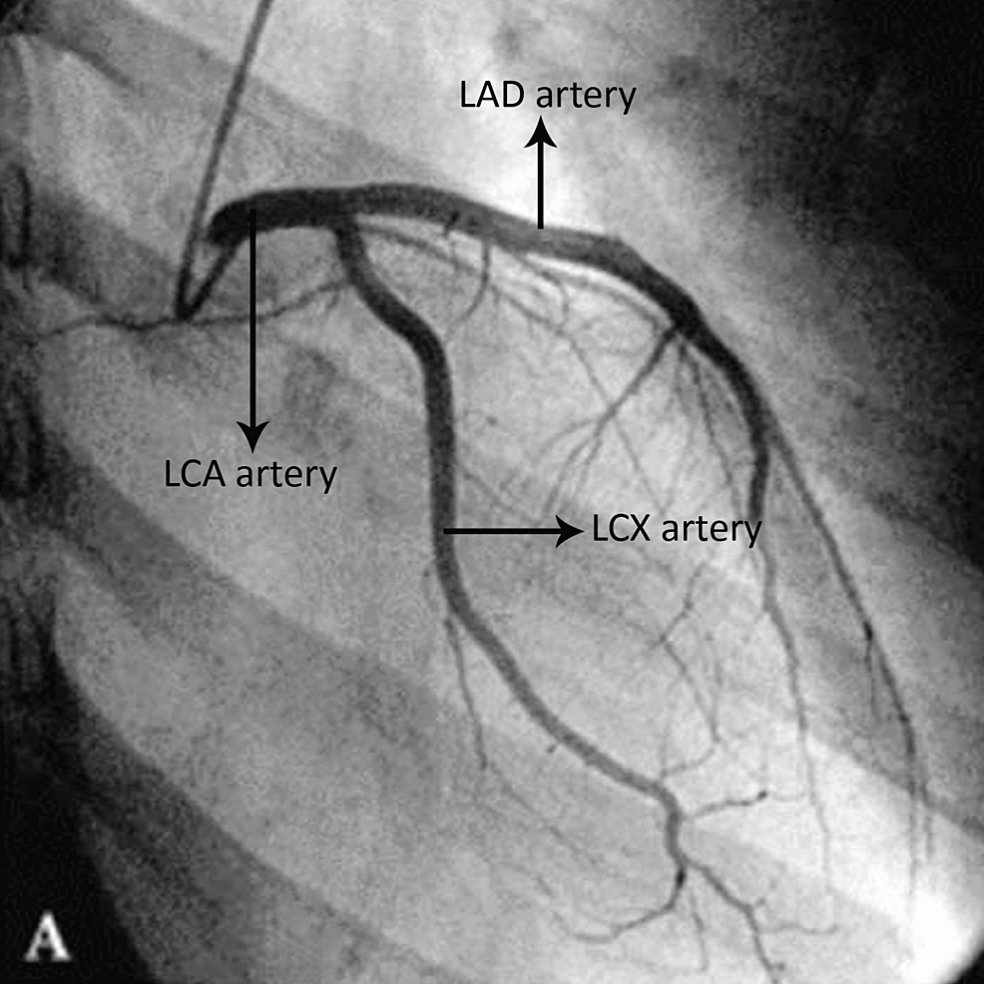
Anteroposterior caudal view of coronary angiogram showing LAD, LCX, and LMCA LAD: left anterior descending, LCX: left circumflex, LCA: left main coronary artery (LMCA) The figure is from the angiograms of patients included in the current study

**Figure 2:**
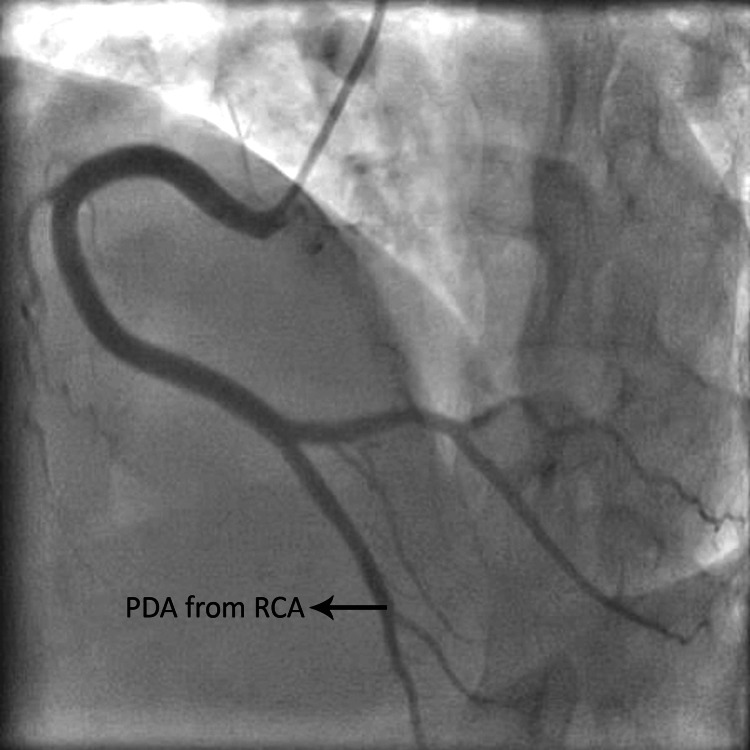
Left anterior oblique view of coronary angiogram showing right dominance RCA: right coronary artery, PDA: posterior descending artery The figure is taken from angiograms of patients included in the current study

**Figure 3:**
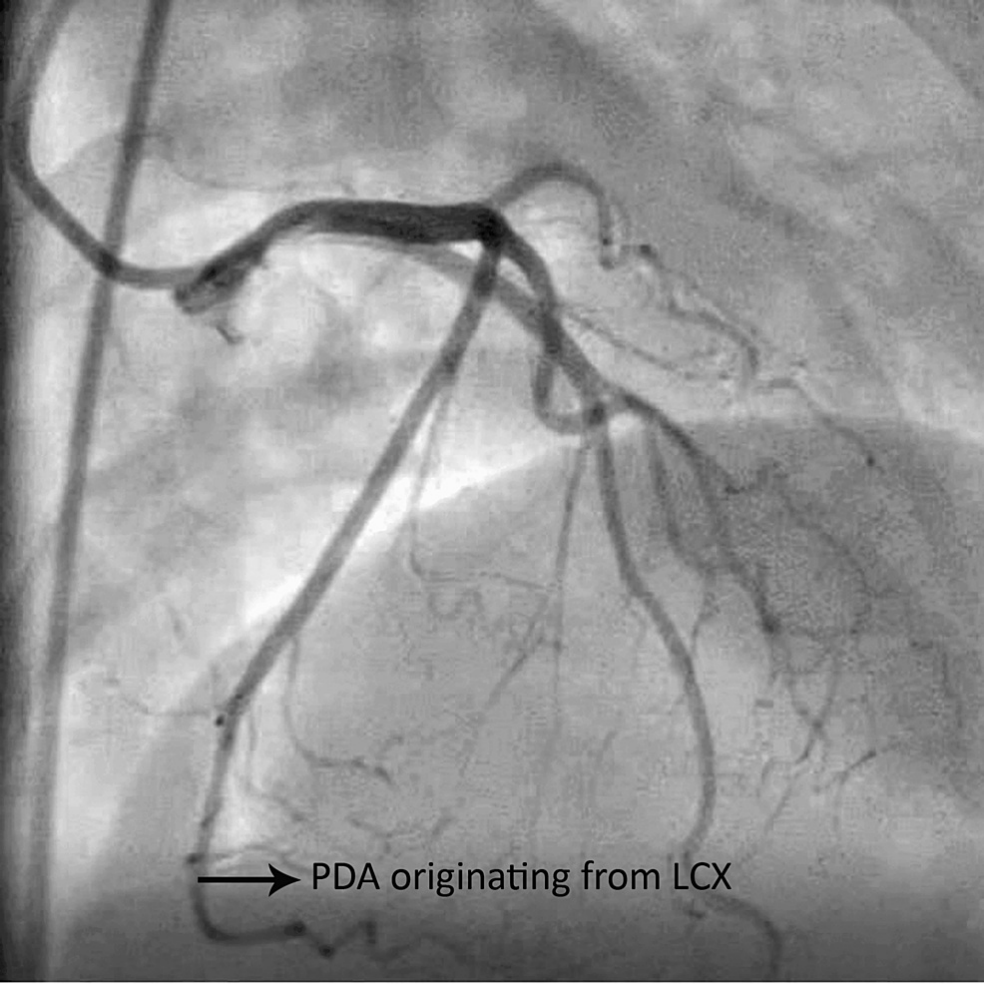
Anteroposterior cranial view of coronary angiogram showing left dominance PDA: posterior descending artery, LCX: left circumflex The figure is from angiograms of patients included in the study

**Figure 4:**
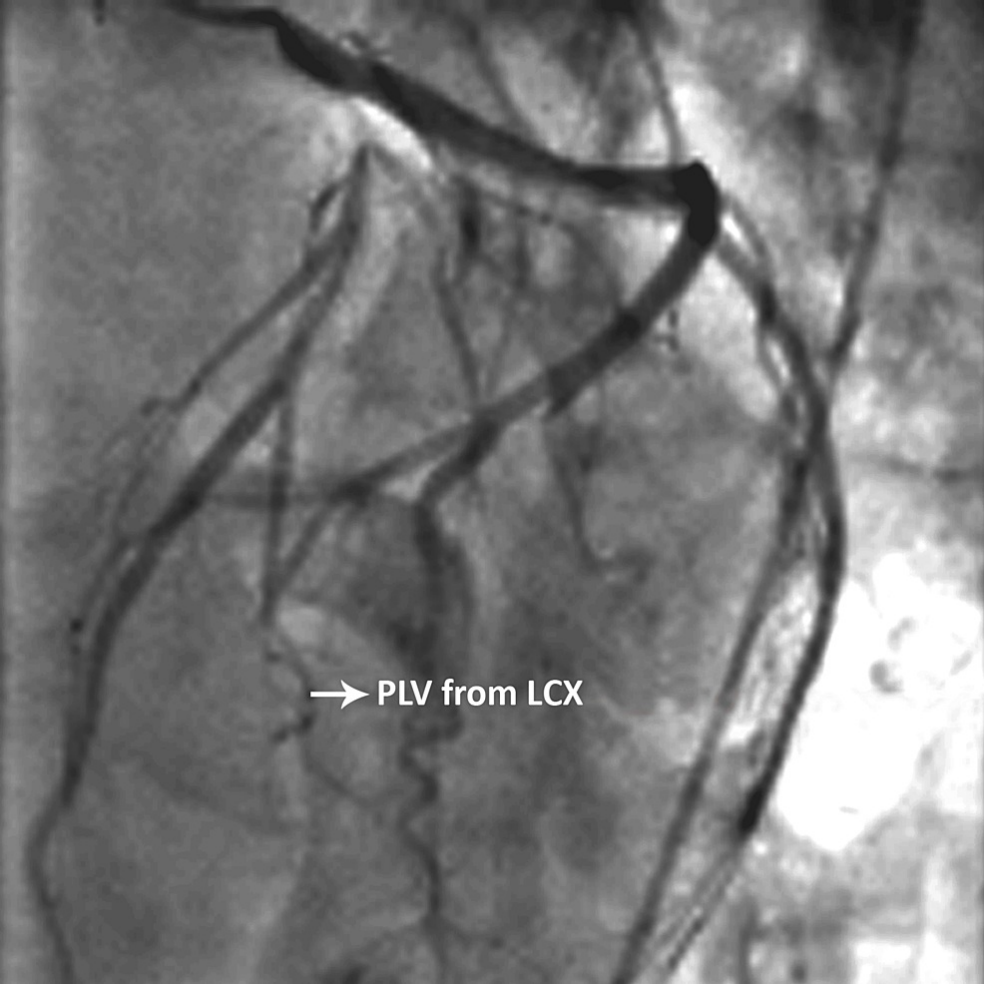
Left anterior oblique cranial view of coronary angiogram showing PLV from LCX PLV: posterior left ventricular, LCX: left circumflex The figure is from angiograms of patients included in the current study

The mean age of the study participants was 54.66±10.66 years. The mean indexed luminal diameter among the 196 females was LMCA (3.45±0.53), LAD (3.46±0.55), LCX (3.24±0.54), and RCA (3.08±0.59) and for the 257 males was LMCA (3.70±0.60), LAD (3.54±0.48), LCX (3.36±0.58), and RCA (3.25±0.62), as shown in Table 1. Males had a statistically significant larger coronary artery diameter for LMCA, RCA, and LCX compared to females. The size difference in the case of LAD was not significant (Figures 1-4 ). When males and females were considered together, LMCA was found to be the largest coronary artery, followed by LAD, LCX, and RCA (Table 2).

**Table 1:** Effect of gender on coronary artery luminal diameter (male 257, female 196) RCA: right coronary artery, LMCA: left main coronary artery, LAD: left anterior descending, LCX: left circumflex

Coronary artery	RCA		LMCA		LAD		LCX	
Gender	Male	Female	Male	Female	Male	Female	Male	Female
Diameter (mean±SD) mm	3.25±0.62	3.08±0.59	3.70±0.60	3.45±0.53	3.54±0.48	3.46±0.55	3.36±0.58	3.24±0.54
p-value	0.004		0.000		0.093		0.026	

**Table 2:** Mean diameter of coronary arteries in the total study population of 453 (male 257, female 196) LMCA: left main coronary artery, LAD: left anterior descending, RCA: right coronary artery: LCX: left circumflex

Coronary arteries	LMCA	LAD	LCX	RCA
Diameter (mean±SD) mm	3.59±0.58	3.50±0.62	3.31±0.57	3.18±0.57

Of the 453 participants, 387 (85.43%) had right coronary dominance which, when gender sub-classified, showed 222 (57.36%) males and 165 (42.64%) females. Left coronary dominance was seen in 48 (10.59%) of which 23 (47.92%) were males and 25 (52.08%) were females. The co-dominant pattern of coronary artery stood at 18 (3.97%) with 12 (66.67%) males and 6 (33.33%) females. The relation between the luminal diameter of coronary arteries and the dominance pattern is tabulated in Table 3. In males, LMCA is significantly larger in all types of coronary circulation, whereas in females, LMCA is larger in all except in the co-dominant pattern. A statistically significant increase in luminal diameter of LMCA was noted in females with advancing age (Table 4).

**Table 3:** Effect of dominance on coronary artery diameter RCA: right coronary artery, LMCA: left main coronary artery

Dominance pattern	Total number of participants	%	Gender	Number of participants	%	Diameter (mean±SD) mm		p-value
						RCA	LMCA	
Right	387	85.43	Male	222	57.36	3.32±0.59	3.51±0.47	0.000
			Female	165	42.64	3.15±0.56	3.45±0.54	0.000
Left	48	10.59	Male	23	47.92	2.61±0.62	3.62±0.49	0.000
			Female	25	52.08	2.64±0.66	3.54±0.66	0.000
Co-dominance	18	3.97	Male	12	66.67	3.24±0.69	3.97±0.53	0.022
			Female	6	33.33	3.16±0.40	3.33±0.40	0.175

**Table 4:** Effect of increasing age on coronary artery luminal diameter LMCA: left main coronary artery, LAD: left anterior descending, LCX: left circumflex, RCA: right coronary artery

Gender	Coronary arteries	Age groups			p-value	
		20-45 years	46-70 years	>70 years		
		Diameter (mean±SD) mm	Diameter (mean±SD) mm	Diameter (mean±SD) mm		
Female	RCA	3.04±0.69	3.06±0.58	3.38±0.47	0.171	
	LMCA	3.22±0.41	3.46±0.54	3.87±0.69	0.004	
	LAD	3.29±0.57	3.45±0.52	3.61±0.58	0.220	
	LCX	3.17±0.51	3.23±0.54	3.50±0.50	0.184	
Male	RCA	3.40±0.54	3.21±0.64	3.14±0.64	0.111	
	LMCA	3.44±0.36	3.57±0.50	3.54±0.60	0.189	
	LAD	3.69±0.52	3.73±0.63	3.46±0.44	0.203	
	LCX	3.33±0.62	3.37±0.57	3.33±0.65	0.869	

## Discussion

The luminal size of a coronary artery is a direct predictor of CAD. It is highly variable in a normal population [10]. Extensive work on coronary artery anatomy has been done in various populations. Genetic and environmental factors, including age, sex, ethnicity, body surface area, and mass of the heart, are correlated with coronary artery diameter [11]. Pre-ascertaining coronary artery size will help in the choice of size of the balloon or stent to be used in PCI [12].

The present study showed that LMCA is the largest coronary artery in the study population, followed by LAD, LCX, and RCA. Studies in the Indian population by Raut et al. [13] and in the Iraqi Kurdish population by Imad et al. [14] also observed that the LMCA is the largest coronary artery. Parallels can also be drawn between the present study and that of Kaimkhani et al. [15].

Influence of gender on coronary artery diameter

In the present study, we found a relationship between gender and coronary artery diameter. Except for LAD, all other coronary arteries are significantly larger in males compared to females. While Mahadevappa et al. reported comparable findings, the observed disparity did not reach statistical significance when normalized to body surface area [16]. Imad et al. also noted a significant difference in coronary artery size between males and females, specifically in terms of LMCA, LAD, and LCX. However, no statistically significant variance was observed in the proximal RCA [14]. According to Dickerson et al., the female gender predicted a smaller diameter at the proximal part of LAD and RCA [17]. In their study, Hiteshi et al. reported that females exhibit smaller coronary arteries, and this reduced size is not associated with body habitus or left ventricular mass [18].

Women experience a higher prevalence of recurrent angina after CABG in comparison to men, and this has been attributed to poorer coronary artery revascularization [19]. Furthermore, morbidity and early mortality rates following myocardial infarction (MI) and CABG are elevated among women [20]. Dodge et al. identified that women exhibit a smaller epicardial arterial diameter than men [11]. The precise cause of gender-related differences has not been elucidated anywhere. Investigations into the vascular effects of exogenous sex hormone administration have been conducted in small cross-sectional studies in men receiving high-dose estrogens and women receiving high-dose androgens. Males administered with pharmacological doses of estrogens exhibited a significantly smaller brachial artery diameter, whereas women taking high-dose androgens had a significantly larger diameter [20,21]. Variations in the size of coronary arteries between men and women cannot be solely attributed to differences in body sizes. Herity et al. corroborated this observation in their study [22].

Influence of age on coronary artery diameter

The present study demonstrates a statistically significant increase in the diameter of LMCA with advancing age in females, suggesting age-related arterial dilatation. This finding aligns with observations made by Herity et al. in post-mortem studies, where an increase in coronary artery dimension was noted with age [22]. In contrast, Dodge et al. reported no age-related change in lumen caliber [11]. Similarly, post-mortem and angiographic studies conducted by Hutchins et al. and Arnett et al. showed no observable changes [12,23]. The reliability of estimating luminal diameter through the study of postmortem or dissection specimens is subject to question, as it can be influenced by factors such as procurement, preservation, fixation, and the analysis of epicardial coronary arteries [16].

Influence of dominance on coronary artery diameter

Cardiac dominance is contingent on the origin of the posterior interventricular artery which can arise either from the right or left coronary artery. The dominant pattern of the heart has great clinical implications. The dominant RCA plays a crucial role in supplying the atrioventricular (AV) node, occlusion of which can result in an inferior wall infarction, potentially leading to various degrees of AV blocks.

The relationships between coronary artery dominance and valvular heart diseases, as well as valvular replacement surgery, have been elucidated in numerous studies [24]. Makarovic et al. further confirm that the type of coronary artery dominance influences the occurrence and outcome of non-obstructive CAD [25].

In the current study, a right-dominant pattern was predominant at 85.43%, surpassing the left-dominant pattern at 10.59%. A smaller percentage (3.97%) exhibited a co-dominant pattern. Kiran et al., in their study involving 75 hearts, reported a prevalence of 77.33% for right dominance, 18.67% for left dominance, and 4% for co-dominance. However, they found no significant relationship between luminal diameter and dominance [26].

While numerous studies exist on dominance patterns, limited information is available regarding the influence of dominance on the lumen caliber of coronary arteries. Right coronary arteries are significantly smaller in left dominant hearts, according to Dodge et al. He further states that in right-dominant patterns, the diameter of the LCX is smaller [11].

We made an effort to comprehend and explore this influence of dominance. In the current study, both in right and left dominant hearts, across both sexes, the proximal LMCA was found to be significantly larger than the proximal RCA. In cases of co-dominance, males exhibited a significantly larger proximal LMCA compared to proximal RCA, while no significant difference was observed in females.

Limitations

In the current study, coronary artery diameter was not adjusted for body surface area, despite some studies suggesting a relationship between body surface area and lumen diameter.

## Conclusions

In our present study, LMCA emerged as the largest among the coronary arteries. Gender is a significant factor influencing coronary artery diameter. Notably, our study revealed a gradual increase in the diameter of the LMCA with advancing age in females. The precise understanding of normal coronary artery size and its modulation by gender, age, and dominance holds immense value in assessing coronary arteries and optimizing the success of interventional procedures. Our study underscores the potential variations in coronary artery diameter within a population, emphasizing the importance of approaching interventional procedures with a conscious awareness of these factors.
